# Unilateral surgery for pediatric sensory exotropia: clinical characteristics and surgical results

**DOI:** 10.1186/s12886-022-02722-2

**Published:** 2022-12-22

**Authors:** Heba M. Shafik, Mohamed Ashraf Eldesouky, Molham A. Elbakary, Hazem A. Elbedewy

**Affiliations:** Department of Ophthalmology, Faculty of Medicine, Tanta University, El Geish Street, Tanta, Gharbeya Governorate Egypt

**Keywords:** Sensory Exotropia, Supermaximum recession resection, Lateral rectus tendon elongation, Anisometropia

## Abstract

**Background:**

To delineate the clinical characteristics and surgical outcomes of large angle sensory exotropia in pediatric patients.

**Methods:**

The medical records of 54 large angle exotropia ≥40 PD patients aged from 1 to 18 years who were operated on between 2018 and 2021 and were followed up for 1 year were reviewed and contacted. Clinical characteristics and surgical outcomes were analyzed retrospectively. Patients were divided into two groups, group S patients had supermaximum recession resection and group E had augmented recession by lateral rectus muscle elongation with an autograft from the resected medial rectus muscle in the same eye. The clinical characteristics and results of both groups were compared.

**Results:**

The mean age of the studied patients with sensory exodeviation at the time of surgery was 8.3 ± 4.2 years. Mean of the duration of exotropia was 6.9 ± 2.2 years, and the mean of postoperative follow-up was 14.3 ± 4.2 months. Surgical success was achieved in 73.07% of group S and 82.14% of group E. Recurrence was more common with anterior segment pathology. Larger post-operative distant angles were strongly related to poorer visual acuities *P* = 0.001 and not related to the age of onset or the duration. Narrowing of the palpebral fissure improved in both groups at the last follow up *P* = 0.336. The limitation of abduction in both groups improved in the last follow up *P* = 0.145.

**Conclusion:**

The outcome of monocular surgery for sensory exotropia in children is satisfactory with no significant differences in results between lateral rectus muscle tendon autograft elongation technique and supermaximum recession resection. Recurrence is more common with anterior segment pathology. Larger post-operative distant angle of deviation is strongly related to poorer visual acuity.

**Clinical trial registration:**

This study was retrospectively registered at clinicaltrials.gov (ID: NCT04286945) on 25-2-2020.

## Background

Sensory exotropia is a unilateral divergent misalignment of the eyes. It is seen in 5–9% of strabismic patients resulting from loss of vision or longstanding poor vision in one eye [1]. The condition is usually caused by anisometropic amblyopia or an organic disease, such as optic nerve or retinal abnormalities, corneal opacity and cataract. It emerges from an underlying sensory deficit followed by partial or complete disruption of fusion [2]. The angles are characteristically large, ranging from 30 to 100 prism diopters (PD) and increase gradually over time [3]. An angle of ≥40 PD was selected [4], as the cutoff for defining large-angle strabismus. Sensory exotropia is more common than sensory esotropia [5]. In cases of large angle exotropia, three or four muscle surgeries on both eyes are usually a reasonable option [6]. But there is usually a strong preference for a monocular procedure to avoid the exposure of the dominant eye to the risks of surgery [5]. Furthermore, this may preserve some muscles for further intervention in case of recurrence. It also reduces surgical time [4, 7–9]. Although supermaximum recession-resection surgery has the advantage of a more stable alignment, but it also may cause limited abduction, narrowing of the palpebral fissure, and enophthalmos [10, 11]. As an alternative, elongation of the lateral rectus muscle using the resected part of the medial rectus muscle as an autograft was biocompatible, safe and preserve comitancy with less economic burden [12–14].

However, there are few reports regarding characteristic features of pediatric sensory exotropia [15–19].

Here in, we report the causes and characteristic features of pediatric sensory exotropia treated by two techniques for monocular surgery in a tertiary eye hospital in Egypt.

## Methods

**Fig. 1:**
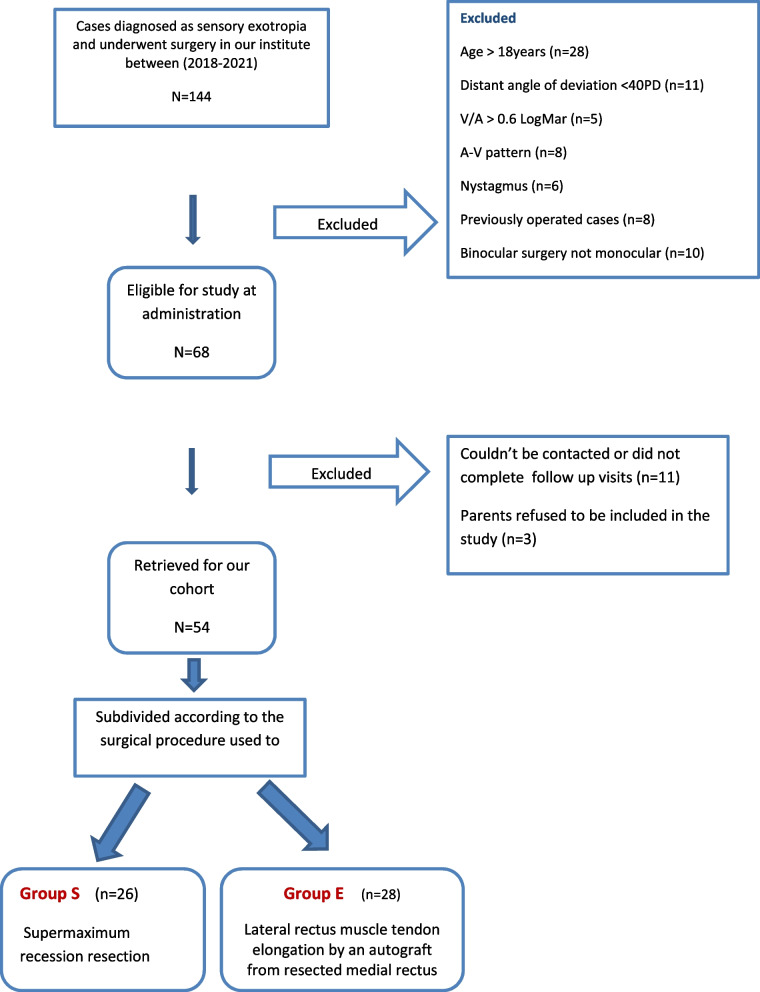
Flow chart for the selection criteria of the studied cases

We conducted a retrospective review of the medical charts of 144 patients with sensory exotropia who were presented to the ophthalmology clinic in a tertiary eye hospital in the period between August 2018 and July 2021. The study included all patients with established diagnosis of sensory exotropia of ≥40 prism diopter (PD) aged from 1 to 18 years, who underwent monocular surgery for the affected eyes with minimum reported follow-up of 12 months and collected complete preoperative and postoperative data including the underlying causes, the age of onset and the duration of visual impairment, the visual acuity of the non-fixing eyes converted to LogMAR, as well as the distant angle of deviation measured by krimsky test in front of the better seeing eye. Recorded post operative angle measurements for near and far distance and recorded postoperative restrictions of abduction if present were scaled from − 4 to 0; with-4 if no abduction beyond midline, − 3 if a75% deficit,-2 if a 50% deficit,-1 if a 25% deficit, and 0 if full ductions. The selection criteria are shown in as a flow chart in Fig. 1.

**Fig. 2:**
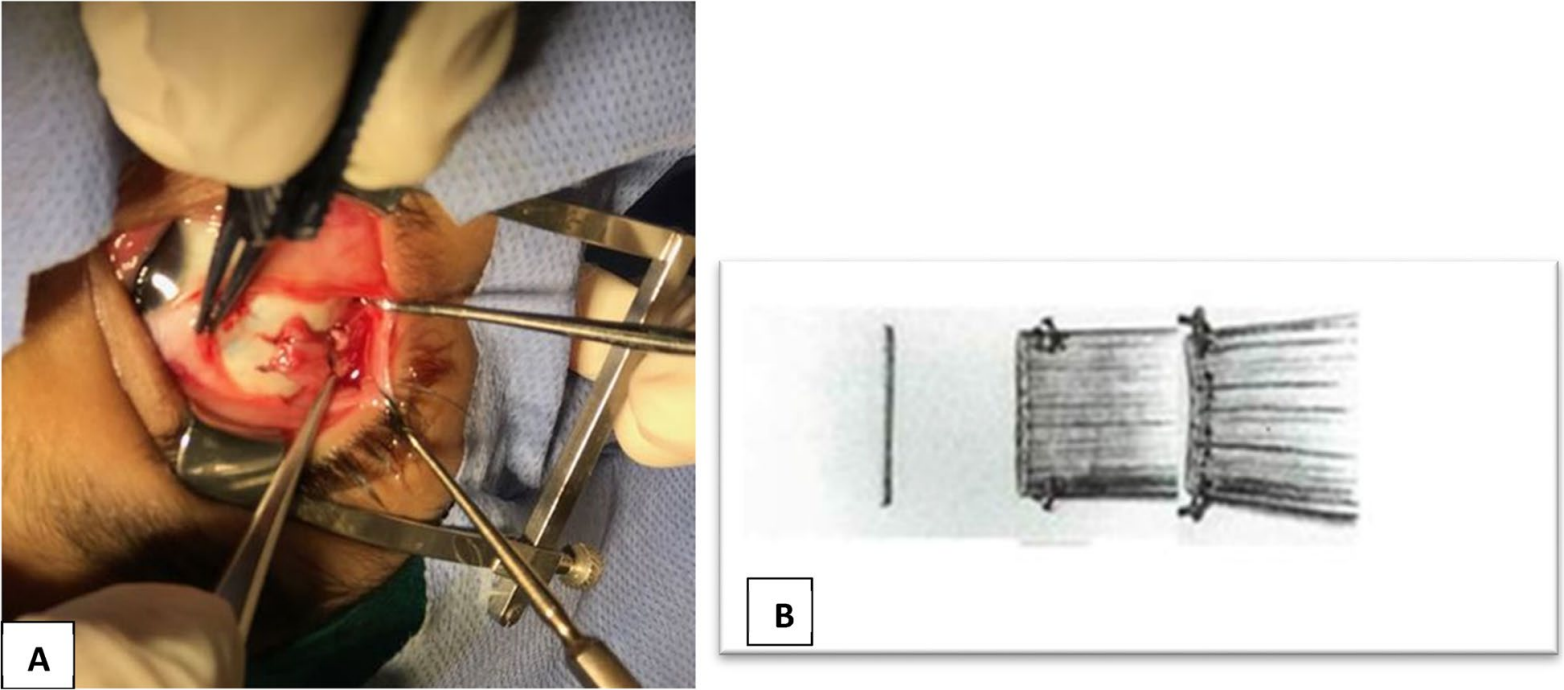
**A** Intraoperative photo showing the recessed lateral rectus muscle after elongation with the autograft from resected medial rectus muscle. **B** Demographic illustration of the mattress sutures taken between the collected autograft and the lateral rectus muscle to augment the recession

We retrieved 68 patients with sensory exotropia who underwent monocular surgery for the affected eye by the same surgeon (H.Sh). Each patient in the list was contacted to return for re-evaluation of motility restrictions and stability of the alignment. We were unable to contact 11 patients and 3 patients refused to be included in this study. The final cohort included 54 patients with a follow-up period ranged between 12 and 18 months. We categorized the patients into two groups: group S included patients who had supermaximum recession resection procedure based on the largest angle of preoperative deviation measured at distance or near. Table 1 provides the formula used for the surgical procedures according to the surgeon’s experience. Group E who had augmented recession by lateral rectus muscle elongation with an autograft from the resected medial rectus muscle in the same eye. The medial rectus muscle was resected first after dissection with a pair of Vicryl 6/0 stitches taken for marking the tendon anteriorly near the insertion. A pair of 6/0 Vicryl sutures were placed near the insertion of the lateral rectus muscle which is incised anterior to the marking stitches and the resected medial rectus muscle tendon is sutures to the lateral rectus muscle by mattress sutures. Now, the elongated lateral rectus muscle is then recessed to the desired amount according to the exotropia distant angles. The amount of resection of medial rectus muscle ranged from 6 to 8 mm as shown in Fig. 2A, B. There was shrinkage in the resected segment of 1–2 mm. The elongated lateral rectus muscle recession did not exceed 7 mm to avoid limitations in abduction. Successful motor alignment was defined as orthotropia or ≤ 10 prism diopter (Δ) exotropia or esotropia at 6 m with spectacle correction worn.

**Table 1:** Surgical dosage table according to surgeon experience in group S

Distant angle measurement in PD	Lateral rectus recession in mm	Medial rectus resection in mm
40	8	5
45	8	6
50	9	6
55	10	7
60	10	8
70	11	8
80–120	12	8–10

The primary outcome measures were the post-operative distant angle measurements in both groups and the rate of recurrence and related factors. The secondary outcome measures were the limitation of abduction and palpebral fissure narrowing in both groups.

### Statistical analysis

All statistical analyses were performed with the Statistical Package for Social Sciences version 23.0 for Windows (SPSS Inc., Chicago, IL). Quantitative data were expressed as mean ± standard deviation (SD). Qualitative data were expressed as frequency and percentage.

A paired t-test was used to compare continuous variables for the mean angle of deviation before and after surgical correction.

Chi-square (X2) test of significance was used in order to compare proportions between two qualitative parameters. A *P* value of ≤0.05 was considered significant.

## Results

**Fig. 3:**
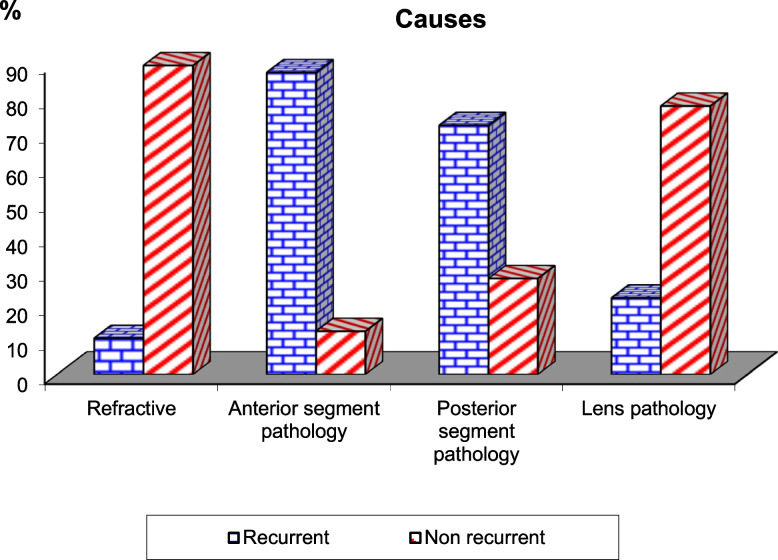
Comparison of the percentage of recurrence in each etiological group after 18 month. Recurrence rate was higher in the anterior segment pathology group and least with the refractive group

The study included 54 patients who met the inclusion criteria, demographic data and clinical characteristics were recorded in Table 2. There was no significant difference between the success rate in both groups after 6 month and after 18 month p = (0.610,0.423) with lower range of distant exotropia angle measurement in group E than group S p = (0.288,0.764) after 6 month and after 18 month respectively as shown in Table 3. Recurrence was more common with anterior segment pathology especially corneal lesions in the studied cases as shown in Fig. 3.

**Table 2:** Demographic and clinical data of the studied cases

Parameter	Group S	Group E	***P*** value
**Number**	26 (48.14%)	28 (51.85%)	0.523
**Sex: Female/Male**	16/10	15/13	0.554
**Laterality: Rt/Lt**	8/18	12/16	0.358
**Age groups at time of surgery:**			
**Below 4 years**	4	0	0.062
**From 4 to 10 years**	10	9	
**From 10 to 18**	12	19	
**Duration of exotropia**	(1–10)years 6.92 ± 2.24	(4–12)years 7.64 ± 2.23	0.243
**VA in affected eye in LogMar:**			
**Can’t Be tested**	4	1	0.122
**Less than 1.0**	14	22	
**More than 1.0**	8	5	
**Preoperative distant angle measurement (PD):**			
**40 to < 80**	12	10	0.435
**80–120**	14	18	
**Causes of exotropia according to etiology:**			
**Anterior segment and lens pathology**	11	11	0.589
**Posterior segment pathology**	8	12	
**Refractive (anisometropia)**	7	5	
**Amount of surgery**			
**Lateral rectus recession(mm)**	8–12 9.04 ± 2.28	4–7 5.96 ± 1.40	0.001*
**Medial rectus resection(mm)**	5–10 7.73 ± 2.04	6–8 6.86 ± 0.85	0.626

* Stands for significant *p* value

**Table 3:** The postoperative clinical features (Distant angle of deviation measurement and success rate) at short (6th month)- and long-term follow-up (18 months)

Outcome features	Group S (***n*** = 26)	Group E (***n*** = 28)	***P*** value
**Distant angle measurement at the 6th month follow up**			0.288
**Mean ± SD (min-max)PD**	−17.43 ± 13.5 (−25–0)	−10.42 ± 8.63 (−20–5)	
**Distant angle measurement at the 18 months follow up**			0.764
**Mean ± SD (min-max)PD**	−20.62 ± 17.15 (−30–0)	− 17.0 ± 9.68 (− 25–8)	
**Success rate at 6 th month**	22/26 (84.62%)	25/28 (89.28%)	0.610
**Success rate at 18 months**	19/26 (73.07%)	23/28 (82.14%)	0.423

X^2^ Chi-Square

**Fig. 4:**
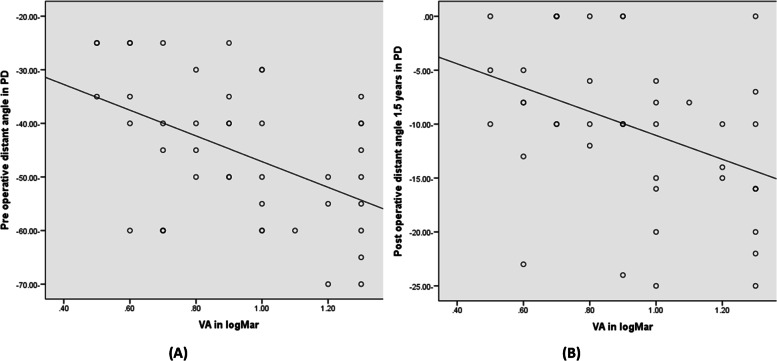
The relation between visual acuity and pre and post angle of deviation after 18 months in all studied case. **A** The relation between visual acuity and pre-operative angle. **B** The relation between visual acuity and post-operative angle measured 18 month after surgery

Larger pre and post-operative distant angles were strongly related to poorer visual acuities (*P* = 0.001*) (Fig. 4A, B), and not related to the age of onset or the duration of exotropia (*p* = 0.393, 0.553) respectively.

Recurrence rate for each etiological group after 18 months was not significant in group S in comparison to group E. As shown in Table 4.

**Table 4:** Recurrence rate for each cause in both groups after 18 month

		Group S		Group E		Total	***P*** value
Causes		Recurrent	Non recurrent	Recurrent	Non recurrent		
**Refractive**	**N**	1	6	0	5	12	0.377
	**%**	1.9%	11.1%	0.0%	9.3%	22.2%	
**Anterior segment and lens pathology**	**N**	4	7	3	8	22	0.647
	**%**	7.4%	13.0%	5.6%	14.8%	40.7%	
**Posterior segment pathology**	**N**	2	6	2	10	20	0.648
	**%**	3.7%	11.1%	3.7%	18.5%	37.0%	
**Total**	**N**	7	19	5	23	54	0.423
	**%**	13.0%	35.2%	9.3%	42.6%	100.0%	

***N*** Number of patients

**Fig. 5:**
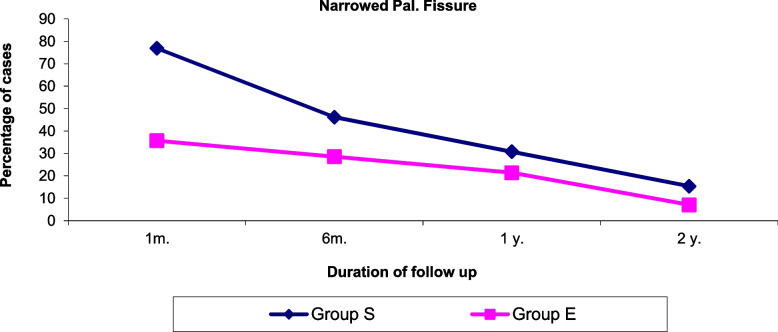
Comparison between the percentage of patients who suffered from narrowing of the palpebral fissure in the 1st month, 6th month, 12 month and after18 month in both groups

There was a significant difference in the number patients who suffered from narrowed palpebral fissure in both groups in the 1st month with more narrowing in group S patients (*p* = 0.002), that improved in the 6^th^month, 12 months and 18 months with no significant difference between both groups. Figure 5, Table 5.

**Table 5:** Number of patients suffering from narrowing of the palpebral fissure after 1 month, 6 month, 12 months, and 18 months in both groups

Narrowing of the palpebral fissure	Group S		Group E		***P*** value
	N	%	N	%	
1 month	20	76.9	10	35.7	0.002*
6 months	12	46.2	8	28.6	0.181
12 months	8	30.8	6	21.4	0.434
18 months	4	15.4	2	7.1	0.336

*N* Number of patients

* Stands for significant *p* value

**Fig. 6:**
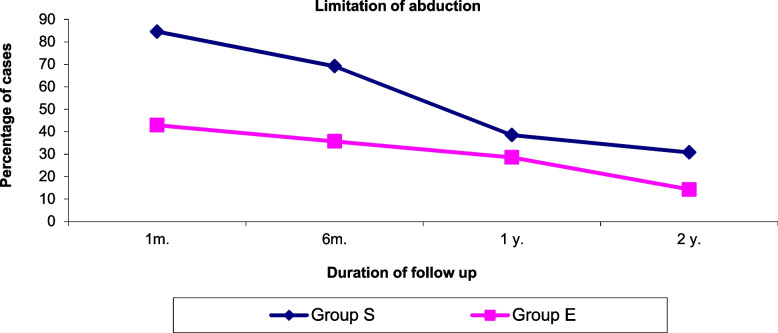
Comparison between the percentage of patients who suffered from limitation of abduction in 1st month, 6th month, 12 month and after 18 month in both groups

Also, there was a significant difference in the number of patients who experienced limitation of abduction in the 1st and 6th month follow up p = (0.006,0.014) respectively between both groups with more limitation in group S,that improved in 12 and 18 months follow ups with no significant difference in both groups. As shown in Fig. 6, Table 6.

**Table 6:** Number of patients suffering from Limitation of abduction after 1 month, 6 month, 12 months, and 18 months in both groups

Limitation of abduction	Group S (***n*** = 26)		Group E (***n*** = 28)		***P*** value
	N	%	N	%	
1 month	22	84.6	12	42.9	0.006*
6 months	18	69.2	10	35.7	0.014*
12 months	10	38.5	8	28.6	0.441
18 months	8	30.8	4	14.3	0.145

*N* Number of patients

* Stands for significant *p* value

Twenty three patients were satisfied by the results in group S (88.5%) versus 26 (92.9%) patients in group E.

## Discussion

In this study we included 54 patients aged ⦤18 years old with sensory exotropia of different aetiologies who met the inclusion criteria and completed follow ups.

Havertape and coauthors [20] have shown that children who acquire visual loss after 2 to 4 years of age are much more likely to develop sensory exotropia. Studies of patients with unilateral congenital cataracts show an even distribution between esodeviations and exodeviations [21].

In the current study, 73.07% of the patients in group S and 82.14% of group E with sensory exotropia had successful outcome in an average of 1.4 ± 0.4 years of follow-up. These results are consistent with those reported in other studies for sensory strabismus surgery, although the inclusion criteria and the duration of the follow-up vary among different reports [22, 23].

Here in we defined surgical success according to the distant angle of deviation. The goal of surgery was initial orthotropia for distance postoperatively rather than overcorrection at near distance. A lower range of distant angle measurement was detected in group E than group S p = (0.288,0.764) after 6 month and after 18 months respectively.

As regard the etiology of sensory strabismus, Kim and coauthors [5] found that cataract was the most common cause (71.7%) of all patients, followed by optic nerve disorder, retinal detachment, glaucoma and lens subluxation. Sidikaro and von Noorden [18] reported that anisometropic amblyopia was the main cause of sensory strabismus, and that cataract and corneal opacities were less common. However, Havertape et al. [20] found that cataracts were the most frequent cause, followed by optic nerve disorders and anisometropic amblyopia. Choi and coauthors [7] found that the most common cause of vision loss in pediatric sensory strabismus was optic nerve disease. In this study we found that anterior segment pathologies (corneal and lens causes) was the primary cause of sensory strabismus in 22 patients (40.2%), followed by posterior segment pathologies in 20 patients (optic nerve lesions, chorio-retinal lesions) (37%), then anisometropia in12 patients (22.2%). Recurrence in the studied cases after 18 months was more common in anterior segment pathology especially corneal lesions. Also, there was no significant difference between group S and group E in recurrence rate for each etiological group after 18 months. This is explained by the low potentialities of fusion regain in sensory strabismus [2, 5].

Although in large-angle exotropia, a three-muscle surgery is a reasonable option [23–25], but patients suffering from sensory exotropia usually have a strong preference for monocular surgery to avoid surgery on the sound eye [24]. Moreover, several other studies reported that good surgical outcome could be obtained with only two-muscles surgery in large-angle sensory exotropia [24, 26]. Thus, we confined surgery to the amblyopic eye either by supermaximum recession resction procedure in group S or Lateral rectus elongation by an autograft from resected medial rectus muscle in group E.

Preoperative deviation has been proven to be the main factor influencing the outcome in patients suffering from exotropia in several studies [27, 28]. In our studied cases larger preoperative distant angles and poorer results with larger post-operative distant angles were strongly related to poorer visual acuities (*P* = 0.001*), and not related to the age of onset or the duration of exotropia (*p* = 0.393, 0.553) respectively. This agreed with Gusek-Schneider et al. who stated that surgical success in sensory exotropia was correlated to visual acuity and that better visual acuity may predict more maintenance of alignment [28]. Ruttum reported Initial instability in early postoperative alignment for exotropia and suggested that pain, inflammation, blurred vision, and altered muscle dynamics may be the rational and vary greatly among patients [29].

In the present study palpebral fissure narrowing after operation did not persist after the 6th month and was more noted in group S treated by maximum recession- resection with no significant difference between both groups from the 6^th^month till the 18th month. It was significantly associated with larger amounts of medial rectus resection. Chang et al. [6] study coincided with our result, as they reported that the narrowing of palpebral fissure in their study was not disfiguring.

A lateral rectus recession exceeding 7.0 mm to 8.0 mm behind the equator is also said to reduce abduction significantly [28, 30] and causes limitation of ocular rotation [31, 32]. Several previous studies of large-angle exotropia have reported success rates ranging from 72 to 80% in bilateral lateral rectus recessions, without significant abduction limitations [32]. Berland and Wilson [33], also reported a success rate of 80% with a 8 to 9 mm bilateral lateral rectus recession, but with abduction limitations in 30% of the 24 patients studied. In the present study, there was a significant difference in the number of patients who had limitation of abduction in the 1st and 6th month follow up p = (0.006,0.014) respectively between both groups with more limitation in group S, that improved in 1st and 2nd year follow ups with no significant difference in both groups.

However, Rayner and Jampolsky [17] advocated the use of large MR resection and maximum LR recession on the amblyopic eye with large-angle exotropia and they reported that the deficient abduction is an advantage for the prevention of exotropia recurrence. In addition, other studies reported that LR recession beyond the equator results in abduction limitation and incomitancy but were not causing disfigurement [6, 24]. Meanwhile, in patients with stretched sclera as in glaucoma and high myopia, or in cases with previous surgery on the same eye, a very large recession or resection may be difficult. Therefore, a surgical technique is required to weaken the extraocular muscle with preservation of motility of the globe at the same time [12, 34]. Diamond [14] and Amitava [13] published a study about large-angle esotropia management with transplantation of resected lateral rectus muscle. They asserted that muscle transplantation is a safe procedure. They had long term stable results. They also stated that it is a simple and innovative procedure that should be put into consideration for correction of large-angle strabismus [15]. This was also reported in cases of large angles sensory exotropia in 2020 by Shafik et al. [12], that reported a satisfactory outcome as regard alignment and motility by using the resected medial rectus muscle autograft to elongate the lateral rectus tendon and augment recession with a follow-up of 6 months with no adverse effects. This agreed with our present study regarding group E with higher success rate 82.14% after18 months and less limitation of abduction and narrowing of the palpebral fissure than group S especially in early post-operative follow ups.

Despite overcorrection and recurrence, surgery for sensory exotropia may produce satisfactory results for many patients. However, results were variable [1].

In the present study we report 88.5% satisfaction in group S and 92.9% patients in group E after 18 months.

This study is limited by its retrospective nature. Also, there could be a selection bias, as we included only patients with follow up for at least 12 months, that is, patients with successful or poor results may not have been contacted or returned to the clinic.

## Conclusion

The long-term outcome of monocular surgery for pediatric sensory exotropia is satisfactory with more favorable outcome regarding early post-operative success, limitation of abduction and less narrowing of palpebral fissure in lateral rectus muscle tendon elongation by autograft from resected medial rectus muscle technique than supermaximum recession resection with no significant difference between both techniques. Recurrence is more common with anterior segment pathology especially corneal lesions. Larger pre and post-operative distant angle of deviation are strongly related to poorer visual acuity.

## Data Availability

The datasets used during the current study are available from the corresponding author on reasonable request.

## References

[1] Dawson EL, Sainani A, Lee JP (2005). Does botulinum toxin have a role in the treatment of secondary strabismus. Strabismus.

[2] VonNoorden GK (1990). Binocular vision and ocular motility: theory and management of strabismus.

[3] Kraft SP, Rosenbaum AL, Santiago AP (1999). Selected exotropia entities and principles of management. Clinical strabismus management: Principles and surgical techniques.

[4] Millan T, de Carvalho KM, Minguini N (2009). Results of monocular surgery under peribulbar anesthesia for large-angle horizontal strabismus. Clinics.

[5] Kim IG, Parks M, Lee SJ (2012). Factors Associated with the Direction of Ocular Deviation in Sensory. Horizontal Strabismus and Unilateral Organic Ocular Problems. Korean J Ophthalmol.

[6] Chang JH, Kim HD, Lee JB, Han SH (2011). Super maximal Recession and Resection in Large-Angle Sensory Exotropia Korean. J Ophthalmol.

[7] Choi MY, Hwang JM (2005). Clinical analysis of sensory strabis¬mus with organic amblyopia in children. J Korean Oph-thalmolSoc.

[8] Govindan M, Mohney GB, Diehl NN, Burke JP (2005). Incidence and types of childhood exotropia: a population-based study. Ophthalmology.

[9] Yoon KC, You IC, Park YG (2002). Clinical analysis of sensory strabismus. J Korean OphthalmolSoc.

[10] Raab EL (1979). Unilateral four-muscle surgery for large-angle exotropia. Ophthalmology..

[11] Rathod D, Goyal R, Watts P (2011). A survey of the management of globe perforation during strabismus surgery in the United Kingdom. Strabismus..

[12] Shafik HM, Eldesouky MA, Tadros D (2020). Lateral Rectus Muscle Tendon Elongation by an Auto Graft from the Resected Medial Rectus Muscle as a Monocular Surgery for Large-Angle Sensory Exotropia. Clin Ophthalmol.

[13] Amitava AK, Goswami AK, Mishra A (2005). Large-angle strabismus and primary true muscle transplantation. J Pediatr Ophthalmol Strabismus.

[14] Diamond GR (1990). True transposition procedures. J Pediatr Ophthalmol Strabismus.

[15] Santiago AP, Ing MR, Kushner BJ, Rosenbaum AL, Santiago AP (1999). Intermittent exotropia. Clinical Strabismus Management: Principles and Surgical Techniques.

[16] Parks MM, Parker JE (1983). editors, Atlas of Strabismus Surgery.

[17] Rayner JW, Jampolsky A (1983). Management of adult patients with large angle amblyopic exotropia. Ann Ophthalmol.

[18] Sidikaro Y, von Noorden G (1982). Observations in Sensory Heterotropia. J PediatrOphthalmol Strabismus.

[19] Mohney BG, Huffaker RK (2003). Common forms of childhood exotropia. Ophthalmology.

[20] Havertape SA, Cruz OA, Chu FC (2001). Sensory strabismus: eso or exo?. J PediatrOphthalmology and Strabismus.

[21] Rosenbaum AL (1993). Exodeviations. Current Concepts in Pediatric Ophthalmology and Strabismus.

[22] Merino P, Mateos C, Gomez De Liano P, Franco G, Nieva I, Barreto A (2011). Horizontal sensory strabismus: characteristics and treatment results. Arch Soc Esp Oftalmol.

[23] Lau FH, Fan DS, Yip WW, Yu CB, Lam DS (2010). Surgical outcome of single-staged three horizontal muscles squint surgery for extra large angle exotropia. Eye..

[24] Chang JH, Kim HD, Lee JB, Han SH (2011). Supermaximal recession and resection in large-angle sensory exotropia. Korean J Ophthalmol.

[25] Chen JH, Morrison DG, Donahue SP (2015). Three and four horizontal muscle surgery for large angle exotropia. J Pediatr Ophthalmol Strabismus.

[26] Thomas S, Guha S (2010). Large-angle strabismus: can a single surgical procedure achieve a successful outcome?. Strabismus..

[27] Gezer A, Sezen F, Nasri N, Gozum N (2004). Factors influencing the outcome of strabismus surgery in patients with exotropia. JAAPOS..

[28] Gusek-Schneider G, Boss A (2010). Results following eye muscle surgery for secondary sensory strabismus. Strabismus..

[29] Ruttum MS (1997). Initial versus subsequent postoperative motor alignment in intermittent exotropia. J AAPOS.

[30] Erkan Turan K, Taylan Sekeroglu H, Sener EC, Sanac AS (2015). Effect of visual acuity on the surgical outcomes of secondary sensory strabismus. Turk J Ophthalmol.

[31] Schwartz RL, Calhoun JH (1980). Surgery of large angle exotropia. J Paediatr Ophthalmol Strabismus.

[32] Celebi S, Kunner AS (2001). Large bilateral lateral rectus recessions in large angle divergence excess exotropia. Eur J Ophthalmol.

[33] Berland JE, Wilson ME, Saunder RB (1998). Results of large (8-9mm) bilateral lateral rectus muscle recessions for exotropia. Binocul Vis Strabismus.

[34] Harrer S, Stangler-Zuschrott E, Rossmann M (1999). Polytetrafluoroethylene in the surgery of cases with severe limitation of abduction; long-term results. Neuro-Ophthalmol..

